# Chemical Composition and Antioxidant Capacity of *Lepidium sativum* Seeds from Four Regions of Morocco

**DOI:** 10.1155/2020/7302727

**Published:** 2020-06-30

**Authors:** Khalid Chatoui, Hicham Harhar, Taha El Kamli, Mohamed Tabyaoui

**Affiliations:** ^1^Laboratoire des Matériaux, Nanotechnologies et Environnement (LMNE), Faculté des Sciences, Université Mohammed V de Rabat, BP 1014 Rabat, Morocco; ^2^Laboratory of Biological Tests, Food and Nutritional Transition Team (ETAN), Ibn Tofaïl University, Kenitra, Morocco

## Abstract

*Lepidium sativum* seeds (*LS*S) from four regions of Morocco have been analyzed for their total chemical composition and antioxidant activities. In the seeds of this plant, the moisture content and yield were, respectively, 9.24–9.88% and 19.13–19.94% of dry weight. Chemical analysis of the seeds revealed amounts of fatty acids, sterols, and tocopherols. The most important fatty acids are linolenic acid (33%) and oleic acid (23%). The main sterol is *β*-sitosterol (50%); the vegetable oil of *Lepidium sativum* revealed an amount of tocopherol (∼1500–1900 mg/kg) with dominance of *γ*-tocopherol. The Folin–Ciocalteu trial evaluated the total phenolic compound, DPPH radical scavenging, ABTS, and chelated iron ions. FRAP measured antioxidant potency. Results indicated that methanol extract from *Lepidium sativum* was a more potent reducing agent and radical scavenger than ethanol extract. Changes in the total phenolic content and antioxidant capacity of *Lepidium sativum* in four different regions grown under normal conditions were evaluated. The antioxidant activity of different extracts was found to correlate significantly with their total phenolic content. These results suggest that *Lepidium sativum* seeds could be used in food supplement preparations or as a food additive, for caloric gain or for protecting against oxidation in nutrient products.

## 1. Introduction

The treatment of diseases from antiquity to the present day has depended in whole or in part on the use of medicinal plants for several reasons, including their action, accessibility, permission, acceptability, and environment [1]. Traditional medicine has a plan for improvement from 2014 to 2023 adapted from the World Health Organization (WHO) [2]. Currently, healing with natural herbal compounds is undergoing a scientific extraction study for screening and chemical identification [3]. Indeed, the medical problems that appear in our body, causing oxidative stress on biomolecules, lead to an imbalance between chemical compounds of plant origin and antioxidants [4]. Among the oxidative, antistress agents that act on reactive species or stimulate the endogenous defence system are vitamins, minerals, phenolic compounds, and carotenoids [5]. The result is a reduction or cessation of diseases such as cerebrovascular risk, diabetes mellitus, arthritis, Parkinson's disease, Alzheimer's disease, and cancer [6].

Morocco has a great diversity of natural and cultivated flora for scientific research in phytotherapy. Thus, some natural products extracted from aromatic herbs and spices are exploited as antioxidants for economic and environmental purposes that could replace the toxic synthetic molecules used recently. This study is based on the seeds of the *Lepidium sativum* (*LS*S) plant, also known as garden cress or garden pepper; it is called “*Hab rchad”* in Morocco. Its seeds contain 27% protein, 14–26% fat, 35–54% carbohydrates, and 8% crude fiber [7]. Carbohydrates of *LS*S include 90% nonstarch polysaccharides and 10% starch [8]. *LS*S contain 20–25% oil, and the main fatty acid is linolenic acid, 32–35%. They also contain natural antioxidants (tocopherols and carotenoids) that help the oil fight rancidity. Imidazole alkaloids, lepidine, monomeric alkaloids, sinapic acid, and sinapine are the most important in *LS*S [9]. It was noted that chemical compounds of plant origin that are studied as secondary metabolite principles are immediately responsible for antioxidant, antimicrobial, antifungal, anticancer, anti-inflammatory, etc. activity [10]. Thus, *LS*S have been studied as potential bioactive sources, and many of them have shown strong antioxidant capacity and high phenolic levels in some regions of Morocco.

However, studies on the antioxidant and bioactive capacity of *LS*S compounds are still scarce, especially considering seeds that are grown in different regions of Morocco, with different climates, characteristics, and geographical conditions. The aim of our research is to determine the variability in the chemical substance of vegetable oil, the total phenolic compounds, and the antioxidant capacity (ABTS, DPPH, and FRAP tests) among *LS*S from four different locations in Morocco.

## 2. Materials and Methods

### 2.1. Plant Material

The collection of *LS*S was carried out in four different Moroccan ecosystems (Table 1), the region of Tafraout (TF) located in southwest Morocco, 158 km from Agadir; El-Haouz (HZ), 44 km from Marrakech; Ben-Ahmed (BA), 86 km from Casablanca; and Rommani (RM), 75 km from Rabat. The seeds were harvested in June 2014. After harvest, the seeds were stored at 4°C until their processing.

### 2.2. Seed Analysis

The percentage by mass of the moisture content of *LS* seeds is revealed by the AOAC 934.06 technique [11] using an oven (VWR, Sheldon Manufacturing, Inc., Cornelius, Oregon, USA), whose temperature has been kept constant at 103 ± 2°C. The oil yield was measured in accordance with DIN EN ISO 659 [12]. Oil extraction was carried out by Soxhlet using hexane as solvent; however, to obtain the alcoholic extracts, the cold maceration method was used, with methanol (MeOH) and ethanol (EtOH) being the two solvents used. All extracted samples were stored at +4°C until use.

The yield is expressed as a percentage and is given by the following formula:

(1) $$\begin{array}{c} \mathrm{yield}\;\left(\%\right)=\frac{\text{mass of oil }\left(\text{or dry extract}\right)}{\text{mass of plant material}}\times 100. \end{array}$$

### 2.3. Analysis of Seed Oils

The fatty acids were analyzed by gas chromatography according to ISO 5508 [13], and the results were expressed as a relative percentage of each fatty acid present in the sample.

The composition of the sterol was measured according to ISO 6799 [14], while the substance of the tocopherols was determined according to ISO 9936 [15].

### 2.4. Total Content of Phenols and Flavonoids

#### 2.4.1. Determination of Total Phenol Content

A mixture of 0.5 mL of extract solution with 2.5 mL diluted Folin–Ciocalteu reagent and 1 : 10 distilled water was made up to 4 mL of 7.5% Na_2_CO_3_, w/v. This was incubated in a water bath at 45°C for 30 minutes, and the OD optical density was read at 765 nm by the UV-Vis spectrophotometer. The standard gallic acid curve was obtained under the same conditions as above using a range of concentrations. The total phenolic compound was measured in gallic acid equivalents (*µ*g gallic acid equivalent GAE/mg extract) [16].

#### 2.4.2. Determination of Flavonoid Content

5% sodium nitrite solution, 0.075 mL, was added to the 0.25 mL extract solution, to which 1.25 mL distilled water was added. The mixture was kept for 5 min, and then 0.15 mL of 10% aluminum chloride was added for 6 min, and 0.5 mL of 1 M sodium hydroxide was added. The mixture was diluted with 0.275 mL distilled water, and the optical density reading was taken at 510 nm relative to a standard curve prepared by quercetin. The flavonoid content was revealed in quercetin equivalent (*µ*g quercetin equivalent QE/mg extract) [17].

#### 2.4.3. Determination of Tannin Content

The absorbance of a mixture of 500 *µ*L of extract solution with 3 mL of 4% vanillin-methanol solution and 1.5 mL hydrochloric acid was measured and left to stand for 15 minutes. The result was given in mg catechin equivalent (*µ*g catechin equivalent CE/mg extract) [18].

### 2.5. Antioxidant Activity

#### 2.5.1. DPPH Free Radical Scavenging Activity

The evaluation of the antioxidant activity of extracts was carried out by the DPPH (1, 1-diphenyl-2-picrylhydrazyl) according to the protocol described by Nounah et al. [19]; a 0.2 mM solution of DPPH was prepared in ethanol, and 0.5 mL of this solution was added to 2.5 mL plant extract and left at room temperature for 30 min, after which DO was read at 517 nm from control samples. The IC_50_ value is used to express the DPPH results and is defined as the amount of antioxidant required to reduce the radical to 50%. It is inversely related to antioxidant capacity. Lower IC_50_ values indicate greater effectiveness of the antioxidant power of the extract.

#### 2.5.2. ABTS Radical Scavenging Test

Stock solutions of 7 mM 2,2-azino-bis(3-ethylbenzothiazoline-6-sulfonic acid) (ABTS) and 2.4 mM of potassium persulfate K_2_S_2_O_8_ in equal volumes were left in the dark for 12–16 h at room temperature. Prior to analysis, the ABTS solution was diluted in ethanol to give an OD of 0.7 ± 0.02 at 734 nm. 2 mL of the resulting solutions was allowed to react with 200 *μ*l plant extracts at different concentrations, the reaction mixture was vortexed, and the OD was measured at 734 nm after 30 min. The same procedure was performed for Trolox at different concentrations. The percentage inhibition of ABTS^+^ by the different extracts was measured and evaluated with Trolox. The inhibition concentration parameter IC_50_ was used to explain the results of the ABTS^•+^ method. The discoloration of the sample was plotted against the sample concentration to calculate the IC_50_ value. It is defined as the amount of sample required to reduce the absorbance of the ABTS^•+^method by 50% [19].

#### 2.5.3. Ferric Reducing Antioxidant Power (FRAP)

Different concentrations of extracts from the stock solution and Trolox standard solution were mixed with 2.5 mL of phosphate buffer (0.2 M, pH 6.6) and 2.5 mL of potassium ferricyanide (1%, w/v).

The mixture was incubated at 50°C for 20 minutes. 2.5 mL of 10% w/v trichloroacetic acid was added to the reaction mixture. It was then centrifuged at 3000 g for 10 min. The supernatant of the 2.5 mL solution was mixed with 2.5 mL of deionized water and 0.5 mL of 0.1% w/v ferric chloride. DO was measured at 700 nm with a reaction time of 30 min. The reducing power of the extracts was represented in Trolox equivalent (*µ*g Trolox equivalent/mg extract) [20].

### 2.6. Statistical Analysis

The analysis of variance (ANOVA) was performed using IBM SPSS Statistics 21 software to test the statistical significance of Tukey tests at a 95.0% confidence level, and the results were presented as means ± standard error of the mean. The Pearson correlation calculation was performed using Microsoft Excel 2010 to estimate the results of the DPPH, ABTS, and FRAP tests obtained for total phenol content (TPC), total flavonoid content (TFC), and total tannin content (TTC).

## 3. Results and Discussion

### 3.1. Extract Moisture and Yield

The results of this analysis revealed a moisture content of less than 10% in the different regions of the *LS*S. The moisture content is almost the same in all four regions: a high moisture content was found in the TF region, 9.88 ± 0.03; the HZ and BA regions have a moisture content of 9.51 ± 0.04 and 9.53 ± 0.10, respectively; and a moisture content of 9.24 ± 0.05 was found in the RM region.

Based on the data recorded in Table 2, the amount of water and volatile matter in the seed exceeds 9% for all four regions, values higher than those proposed by Brooker and Patterson, 8%, for oilseed storage [21, 22].

Lipid was obtained by extraction of *LS*S with hexane using Soxhlet for 8 hours. The results showed that there was no difference in the oil extraction rate. In general, the oil yielded above 19%, with a maximum of about 19.94% for TF and a minimum of 9.13% for RM. All these values were lower than those of Diwakar et al. [23] who found that the total oil content of the solvent extracted from *LS* was 21.54%, and the cold expression was 12.60%. The oil content of *LS*S is partially lower than that of other edible oilseeds such as mustard (25–40%), rapeseed (40–45%), and linseed (40–45%) of the *Cruciferae* family [23].

The methanolic (MeOH) and ethanolic (EtOH) extract were obtained by extraction of *LS*S with methanol and ethanol by hot maceration for 8 hours. The results obtained show a large difference between the yield of MeOH and EtOH extract from the four regions. The TF region was significantly higher than the other regions. The content was 31.9% MeOH and 21.21% EtOH. The lowest yield was recorded in the RM region for MeOH extract, 19.03%, and in the BA region for EtOH extract, 10.56%.

### 3.2. Fatty Acid Composition

Table 3 groups the results obtained from oils that are converted to methyl esters and analyzed by gas chromatography on a capillary column of the four regions.

*LS* oil contains more than 80% of unsaturated fatty acids, which are composed of major components such as oleic, linolenic, and linoleic acid and a minor compound, palmitoleic acid, less than 0.3%. It also contains saturated fatty acids such as palmitic, stearic, and arachidic acid. The fatty acid composition of *LS* reported in this study is consistent with previously reported data [8, 23].

The main fatty acid of LSS (linolenic acid) is one of the essential fatty acids. It is mainly used to treat hereditary or acquired deficiency of the enzyme Δ6-desaturase in humans, mainly in the elderly people and in people with stress, diabetes, or alcoholism [24].

Our scientific research conducted at the four stations showed no significant changes in fatty acid levels. The geographical origin therefore does not change the fatty acid composition. These results confirm that climatic conditions have no influence on the fatty acid composition of *LS* oils from different localities, and are also consistent with the data acquired on the geographical effect on the composition of argan oil [25] and with the study of the effect of geographical origin on the fatty acid composition of olive oils from Italy [26].

### 3.3. Phytosterol Composition

To determine the impact of geographical origin on the sterol fraction, we opted for a GC analysis that led to the results in Table 4.

The sterol fraction of *LS* oil is mainly composed of *β*-sitosterol and campesterol. As is the case for most vegetable oils, *β*-sitosterol was the main phytosterol present in *LSS* oil. This result is in agreement with the data acquired by Moser et al. [27]. The proportions of *β*-sitosterol and campesterol in total sterols vary between 50.17 and 48.17%, and between 22.85 and 24.22%, respectively. These biomolecules provide protection against colon, prostate, and breast cancer [28].

Our results indicate a significant influence (*P* < 0.05) of geographical origin on total sterols in *LS* oil. It ranged from 92.15% in the RM region to 99.58 in the TF region. Thus, the sterol composition of *LS* oil is influenced by its origin. This is consistent with the results of Ben Temime et al., who found that geographical origin and climatic factors influence the sterol composition of olive oils [29].

### 3.4. Tocopherol Content

Analysis of the tocopherol fraction by high-performance liquid chromatography (HPLC) shows a variation of this fraction according to geographical origin. Examination of Table 5 distinguishes essentially four tocopherols, the most important of which is *γ*-tocopherol, followed by *δ*-tocopherol and *α*-tocopherols, while *β*-tocopherol is detected only in the HZ region.

However, the region influences the total tocopherol content, since oils from the HZ region recorded the highest values, 1940.26 mg/kg, followed by oils from the TF region, 1877.8 mg/kg. Oils from the BA region recorded the lowest total tocopherol content at 1510.46 mg/kg. All values are higher than those found by Zia-Ul-Haq et al. [30] for the Pakistani *LS*, 1397.3 mg/kg, but comparable to those found by Moser et al. [27]. For comparison, existing crude vegetable base oils with relatively high levels of *γ*-tocopherol include corn oil (942 mg/kg), soybean oil (273.3 mg/kg), argan oil (626 mg/kg), and cotton oil (387 ppm) [31–33].

Tocopherols have vitamin E activity.This vitamin is a powerful antioxidant that captures free radicals and neutralizes destructive oxidation [34]. The main tocopherol in *LS* oil is *γ*-tocopherol, which is a natural antioxidant. The exceptionally high percentage of *γ*-tocopherol could make watercress oil a potentially useful industrial source of this natural antioxidant.

### 3.5. Total Phenols, Flavonoids, and Condensed Tannins

Polyphenols are highly demanded compounds. Plants rich in polyphenolic metabolites have certain biological activities such as antiviral, antithrombotic, anticarcinogenic, antiallergic, antimicrobial, hepatoprotective, and antihypertensive activities [35, 36].

The determinations of total phenols, flavonoids, and tannins were calculated from the linear regression equation of the calibration curve, using gallic acid, quercetin, and catechin as standards, respectively. It can be seen in Table 6 that the total phenol content of the samples ranges from 52.79 to 94.48 mg GAE/g extract. Among the studied areas, the TF had the highest phenol content, 94.48 mg GAE/g extract, while the lowest proportion was found in the RM, 52.79 mg GAE/g extract. The considerable difference in phenolic content may be due to environmental factors such as maturity period, climate, location, temperature, productivity, diseases, vegetative part, and environmental aggressiveness [37]. In addition, rainfall continues to affect the phenolic content [38]. A positive correlation is recorded between polyphenol and altitude, while a negative correlation is marked between total phenol and precipitation. Increased phenolic content in plants under water was stressed [39].

Almost all samples were found to be rich in flavonoids. The total flavonoid content ranged from 37.63 in TF to 20.04 mg QE/g extract in RM.

This test shows that the methanolic fraction of the BA contains the highest content of condensed tannins, with a value of 31.50 mg CE/g extract. On the other hand, the RM EtOH fraction recorded the lowest level at 8.33 mg CE/g extract. This change is interpreted by the fact that the extraction of condensed tannins depends on the origin of the seeds, the solvent used, and the operating conditions.

### 3.6. Antioxidant Activity

In this study, the commonly accepted tests DPPH, FRAP, and ABTS were used to assess the antioxidant activity of plant extracts. The results of these tests are presented in Table 7 and are an average of three independent measures.

The antioxidant activity of the different extracts from the four *LS* regions was determined by calculating the IC_50_ of the trapping activities of DPPH and ABTS. In parallel, in the FRAP test, the antioxidant activity was determined by calculating the EC_50_ of the FRAP capacity for each extract. The lower the value of the IC_50_ or EC_50_, the higher the antioxidant activity. The DPPH IC_50_, ABTS IC_50_, and FRAP EC_50_ values were compared to the standard ascorbic acid IC_50_ value. The IC_50_ values for DPPH and ABTS trapping activities of different extracts from the four regions of the *LS* were in the range of 119–196.9 and 187.8–456.8 *μ*g/mL, respectively, while the FRAP EC_50_ ranged from 777.0 to 1237.0 *μ*g/mL.

The free radical stability of DPPH is observed by OD at 516 nm. The reduction in absorbance of DPPH is related to the antioxidant potential of a sample. The concentration of a sample or standard that can inhibit 50% of DPPH radical activity is called the IC_50_ of DPPH scavenging activity. For the positive control ascorbic acid, the results showed that both extracts from the four regions have a very significant antioxidant capacity. We also observed that the extract of MeOH had a more powerful antioxidant activity than the extract of EtOH, but all these values were very high compared to the standard. A positive correlation was observed between the DPPH and phenolic compounds assay for both MeOH and EtOH extracts, and all four locations had a high degree of acceptance (*P* < 0.05). This correlation indicated that the richness in phenolic compounds enhances the antioxidant activity of the plant extract.

The radical cation ABTS was produced in stable form using potassium persulfate. After obtaining the stable absorbance, the antioxidant plant extract was added to the reaction medium, and the antioxidant power was measured by studying discoloration. It should be noted that the TEAC data for all plant species obtained by the ABTS test were higher than those revealed by the DPPH test.

As shown in Table 7, the IC_50_ values for ABTS^•+^ radical scavenging activity ranged from 187.8 to 456.8 *µ*g/mL. By comparing these values with those of the standard, it is evident that the samples tested are effective in their ability to remove the ABTS^•+^ radical cation at an average concentration.

*LS* extracts from all four regions showed a high level of significance (*P* < 0.05) between the ABTS^•+^ radical and TPC. The positive and significant correlation between TPC and the antioxidant activity of ABTS reinforces the results observed in the DPPH scavenging method used in this study.

This research is consistent with the hypothesis that an increase in total phenolic compounds will increase the antioxidant activity of extracts, which was previously restored [40].

The FRAP assay measures the reduction potential of an antioxidant that reacts with a ferric tripyridyltriazine complex (Fe^3+^-TPTZ) to produce a colored ferrous tripyridyltriazine (Fe^2+^-TPTZ). Breakage of the free radical chain occurs by the donation of a hydrogen atom. At a low pH of about 3.6, the Fe^3+^-TPTZ complex reduces to the blue-colored Fe^2+^-TPTZ, which has an absorbance value at 593 nm.

The results presented in Table 7 showed that the MeOH extract from the TF region had potent activity, EC_50_ = 777 *µ*g/mL, and standard ascorbic acid, EC_50_ = 44 *µ*g/mL. Therefore, this study showed that high levels of phenolic acid compounds found in the *LS* are the major contributors to antioxidant activity.

The significant correlation between the FRAP test and total phenolic compounds (*r*^2^ = 0.991 and *P* < 0.05) proves that the high antioxidant activity could be related to the high amount of polyphenols in the extracts, which reinforces the results observed in the DPPH and ABTS methods used in this study.

## 4. Conclusion

In conclusion, our study indicates an influence of geographical origin on the total sterols of *Lepidium sativum* oil. It ranges from 92.15 in the RM region to 99.58 mg/kg in the TF region. The region also influences the total tocopherol content; oils from the HZ region had the highest values, 1940.26 mg/kg, while oils from the BA region had the lowest total tocopherol content, 1510.46 mg/kg. However, geographical origin does not influence the fatty acid composition. As regards the phenol content, TF had the highest amount (94.48 mg GAE/g extract), while the lowest proportion was found in the RM region (52.79 mg GAE/g extract). The two extracts MeOH and EtOH from the four regions had a very significant antioxidant capacity. We also observed that the methanolic extract had more powerful antioxidant activity than the EtOH extract. The *LS* extracts from the four regions showed a positive and significant correlation between TPC and the antioxidant activity of DPPH, ABTS, and FRAP.

## Figures and Tables

**Table 1:** Geographical data of *LS*S collection sites.

	TF	HZ	BA	RM
Altitude (m)	993	500	547	306
Average temperature (°C)	16.6	19.9	17.2	17.7
Rainfall (mm)	235	242	401	436

**Table 2:** Raw material characteristics (mean value ± standard deviation).

	TF	BA	RM	HZ
Moisture (%)	9.88 ± 0.03	9.53 ± 0.10	9.24 ± 0.05	9.51 ± 0.04
Yield (%)	19.94 ± 0.00	19.37 ± 0.01	19.13 ± 0.00	19.53 ± 0.002
Methanolic extract (%)	31.90 ± 1.14	19.91 ± 0.54	19.03 ± 1.18	21.28 ± 0.80
Ethanolic extract (%)	21.21 ± 1.01	10.56 ± 0.55	15.61 ± 0.66	17.34 ± 0.67

**Table 3:** Fatty acid composition (%) of *LS*S oil from the four regions studied.

Fatty acid	BA	RM	TF	HZ
Myristic C14 : 0	0.10 ± 0.00^ab^	0.10 ± 0.00^a^	0.10 ± 0.00^a^	0.11 ± 0.00^b^
Palmitic C16 : 0	10.09 ± 0.35^a^	9.98 ± 0.00^ab^	9.86 ± 0.06^b^	9.67 ± 0.00^c^
Palmitoleic C16 : 1	0.21 ± 0.00^a^	0.20 ± 0.00^b^	0.23 ± 0.00^a^	0.23 ± 0.00^a^
Stearic C 18 : 0	3.17 ± 0.03^a^	3.32 ± 0.00^b^	3.37 ± 0.00^b^	3.37 ± 0.00^b^
Oleic C18 : 1	24.08 ± 0.01^a^	23 ± 0.00^b^	23.37 ± 0.00^c^	23.84 ± 0.00^d^
Linoleic C18 : 2	12.20 ± 0.05^ab^	12.09 ± 0.00^a^	12.19 ± 0.00^ab^	12.27 ± 0.00^b^
Linolenic C18 : 3	33.65 ± 0.29^a^	33.07 ± 0.00^b^	33.26 ± 0.00^c^	33.57 ± 0.46^a^
Arachidic C20 : 0	3.34 ± 0.04^a^	3.39 ± 0.00^a^	3.45 ± 0.00^a^	3.72 ± 0.11^b^
Gadoleic C20 : 1	13.08 ± 0.02^a^	12.29 ± 0.00^b^	12.48 ± 0.00^c^	12.94 ± 0.00^d^
Saturated fatty acid	16.72	16.79	16.72	16.87
Unsaturated fatty acid	83.25	80.65	81.53	82.85

The data are the mean of three replicates (*n* = 3e ± SEM); means followed by similar superscript letters in the same row are not different (*P* < 0.05).

**Table 4:** Sterol composition (%) of *LS*S oil in the four regions studied.

Sterol	TF	RM	BA	HZ
Cholesterol	4.00 ± 0.01^a^	2.80 ± 0.00^b^	3.93 ± 0.00^c^	3.99 ± 0.00^a^
Stigmasterol	2.49 ± 0.27^a^	2.13 ± 0.03^b^	2.20 ± 0.00^b^	2.40 ± 0.00^ab^
Campesterol	24.22 ± 0.15^a^	22.85 ± 0.00^b^	23.79 ± 0.05^c^	24.09 ± 0.00^ac^
5-Stigmasterol	4.07 ± 0.03^a^	3.66 ± 0.02^b^	3.60 ± 0.00^b^	3.81 ± 0.00^c^
*β*-Sitosterol	50.17 ± 0.03^a^	48.17 ± 0.00^b^	48.53 ± 0.03^b^	49.49 ± 0.26^c^
5-Avenasterol	13.36 ± 0.32^a^	12.21 ± 0.01^b^	12.66 ± 0.00^ab^	13.46 ± 0.32^a^
7-Stigmasterol	0.30 ± 0.00^a^	0.11 ± 0.00^b^	0.13 ± 0.00^b^	0.20 ± 0.00^c^
7-Avenasterol	0.50 ± 0.01^a^	0.20 ± 0.00^b^	0.31 ± 0.00^c^	0.36 ± 0.00^d^
Total sterol	99.58 ± 0.16^a^	92.14 ± 0.04^b^	95.15 ± 0.10^c^	97.80 ± 0.48^d^

The data are the mean of three replicates (*n* = 3e ± SEM); means followed by similar superscript letters in the same row are not different (*P* < 0.05).

**Table 5:** Tocopherol composition (%) of *LS*S oil in the four regions studied.

	TF	RM	BA	HZ
*α*-tocopherol	0.78 ± 0.00^a^	1.25 ± 0.00^b^	1.69 ± 0.012^c^	0.39 ± 0.00^d^
*β*-tocopherol	00 ± 00^a^	00 ± 00^a^	00 ± 00^a^	1.78 ± 0.00^b^
*γ*-tocopherol	94.48 ± 0.29^a^	94.53 ± 0.016^a^	93.89 ± 0.01^ab^	93.73 ± 0.06^b^
*δ*-tocopherol	4.71 ± 0.04^a^	4.18 ± 0.00^b^	4.29 ± 0.02^b^	3.89 ± 0.01^c^
Total (mg/kg)	1877.8 ± 0.2	1659.9 ± 0.3	1510.4 ± 0.2	1940.2 ± 0.5

The data are the mean of three replicates (*n* = 3e ± SEM); means followed by similar superscript letters in the same row are not different (*P* < 0.05).

**Table 6:** Total contents of phenols, flavonoids, and tannins in the MeOH and EtOH extract from the four regions studied.

		Polyphenols (mg GAE/g extract)	Flavonoids (mg QE/g extract)	Tannins (mg CE/g extract)
TF	MeOH	94.48 ± 1.82^a^	37.63 ± 2.14^a^	26.50 ± 0.07^a^
	EtOH	86.48 ± 0.22^b^	32.51 ± 0.81^bc^	27.79 ± 0.074^b^

HZ	MeOH	83.36 ± 0.98^bc^	33.58 ± 0.33^ab^	25.87 ± 0.072^a^
	EtOH	80.28 ± 0.28^c^	29.24 ± 0.47^c^	26.02 ± 0.31^a^

BA	MeOH	69.46 ± 0.09^d^	24.85 ± 0.48^d^	31.50 ± 0.11^c^
	EtOH	65.15 ± 1.07^e^	23.92 ± 0.64^de^	23.41 ± 0.25^d^

RM	MeOH	59.40 ± 0.62^f^	21.09 ± 0.21^de^	12.85 ± 0.56^e^
	EtOH	52.79 ± 0.30^g^	20.04 ± 0.04^e^	8.33 ± 0.11^f^

The data are the mean of three replicates (*n* = 3e ± SEM); means followed by similar superscript letters in the same row are not different (*P* < 0.05).

**Table 7:** IC_50_ and EC_50_ values of the effective concentration of the DPPH, ABTS, and FRAP free radical scavenging assays for MeOH and EtOH extracts from *LS*S from the four regions.

	DPPH (IC_50_ *μ*g/mL)		FRAP (EC_50_ *μ*g/mL)		ABTS (IC_50_ *μ*g/mL)	
	MeOH	EtOH	MeOH	EtOH	MeOH	EtOH
TF	119.3	134.7	777.0	813.0	187.8	211.3
HZ	143.3	153.8	898.0	947.0	279.0	296.5
BA	162.3	177.4	980.0	1041.0	318.7	339.1
RM	188.0	196.9	1105.0	1237.0	345.0	456.8
Ascorbic A	17.96		44.53		31.47	

## Data Availability

All data supporting the findings are adequately included within the article.
